# Glaucoma and Driving: On-Road Driving Characteristics

**DOI:** 10.1371/journal.pone.0158318

**Published:** 2016-07-29

**Authors:** Joanne M. Wood, Alex A. Black, Kerry Mallon, Ravi Thomas, Cynthia Owsley

**Affiliations:** 1 School of Optometry and Vision Science, Queensland University of Technology, Brisbane, QLD, Australia; 2 Queensland Eye Institute, Brisbane, QLD, Australia; 3 University of Queensland, Brisbane, QLD, Australia; 4 Department of Ophthalmology, University of Alabama at Birmingham, Birmingham, Alabama, United States of America; The University of Melbourne, AUSTRALIA

## Abstract

**Purpose:**

To comprehensively investigate the types of driving errors and locations that are most problematic for older drivers with glaucoma compared to those without glaucoma using a standardized on-road assessment.

**Methods:**

Participants included 75 drivers with glaucoma (mean = 73.2±6.0 years) with mild to moderate field loss (better-eye MD = -1.21 dB; worse-eye MD = -7.75 dB) and 70 age-matched controls without glaucoma (mean = 72.6 ± 5.0 years). On-road driving performance was assessed in a dual-brake vehicle by an occupational therapist using a standardized scoring system which assessed the types of driving errors and the locations where they were made and the number of critical errors that required an instructor intervention. Driving safety was rated on a 10-point scale. Self-reported driving ability and difficulties were recorded using the Driving Habits Questionnaire.

**Results:**

Drivers with glaucoma were rated as significantly less safe, made more driving errors, and had almost double the rate of critical errors than those without glaucoma. Driving errors involved lane positioning and planning/approach, and were significantly more likely to occur at traffic lights and yield/give-way intersections. There were few between group differences in self-reported driving ability.

**Conclusions:**

Older drivers with glaucoma with even mild to moderate field loss exhibit impairments in driving ability, particularly during complex driving situations that involve tactical problems with lane-position, planning ahead and observation. These results, together with the fact that these drivers self-report their driving to be relatively good, reinforce the need for evidence-based on-road assessments for evaluating driving fitness.

## Introduction

Glaucoma is a leading cause of visual field loss in older populations,[1] affecting approximately 60 million people worldwide, with the numbers estimated to increase significantly in the future.[2, 3] Importantly, 20 per cent of glaucoma patients experience progressive visual field loss despite receiving appropriate treatment,[4] and even in developed countries, half of those with glaucoma are unaware that they have the disease.[5]

Driving is a key component in maintaining the independence of older adults, including those with glaucoma. However, older drivers with glaucoma report a number of driving difficulties, including problems with glare, night driving and tasks requiring peripheral vision and visual search,[6] with avoidance of challenging driving situations, [7] and self-reported reductions in their driving ability.[8] Glaucoma is cited as one of the main reasons that older drivers give up driving,[9] with patients having bilateral glaucoma being almost three times more likely to cease driving than those without glaucoma.[10]

A number of studies have reported elevated crash risk in drivers with glaucoma, however, the experimental design of these studies has been of variable quality. Earlier studies reported elevated crash rates in drivers with glaucoma,[11, 12] but did not adjust for confounding factors, while small case control studies have reported increased numbers of simulator and real world crashes in glaucoma patients compared to controls.[13] More recent studies have shown that glaucoma is an important risk factor for self-reported crashes over the previous 10 years [14] and state-recorded crashes,[15–19] however, the underlying reasons for this increased crash risk are unclear. There is also some debate regarding the impact of the location of visual field loss on driving ability and safety, with some studies highlighting the importance of field loss in the lower field [20, 21], while others highlight the importance of field loss in the upper field [19, 22]. These discordant findings may be due to differences in sample characteristics as well as methods of assessing driving ability and safety.

The simulator-based driving performance of persons with glaucoma has been compared to those without glaucoma, involving small numbers of drivers with a variety of levels of glaucomatous visual field loss. One study found that drivers with glaucoma adjusted the position of the steering wheel more frequently (exhibited more jerky steering) than did the controls and were also poorer at detecting letters presented in the driving scene than controls, but there were no other between group differences,[23] while a recent case control study reported that patients with advanced glaucoma had significantly more simulator crashes that were related to reductions in integrated visual field sensitivity.[24] Conversely, a small scale study of drivers with binocular glaucomatous field loss found that a subgroup exhibited safe driving behaviors through increased visual scanning, leading the authors to conclude that binocular field loss doesn’t necessarily impact upon driving safety.[25]

The specific nature of the driving difficulties of those with glaucoma on real roads has not been extensively studied, despite growing evidence that some drivers with glaucoma have an increased risk of motor vehicle collision involvement. The only studies that have assessed on-road driving in drivers with glaucoma have highlighted problems with lane-keeping, negotiating curves and anticipatory skills;[26] and increased driving instructor interventions.[27, 28] However, while providing a useful basis for understanding driving difficulties in glaucoma these studies were limited by relatively small numbers of participants,[26–28] failure to include a control group [26] or assessed limited aspects of driving performance.[27, 28]

The purpose of this study was to investigate which aspects of driving behavior and types of driving situation are most problematic for drivers with glaucoma. Compared to older adults without glaucoma, we hypothesized that lane control and skills involving observation and planning would be impaired in those with glaucoma; we included individuals with mild to moderate glaucomatous field loss, given they are more likely to be actively driving. This type of information regarding specific driving difficulties is essential to provide the basis for the design of interventions to improve the driving ability and safety of this population.

## Materials and Methods

### Participants

Participants with glaucoma were recruited from the clinical records of the Queensland University of Technology (QUT) Optometry Clinic and private ophthalmology practices, and had been diagnosed as having glaucoma by their treating ophthalmologist. The age-matched control participants were recruited as a convenience sample from our existing database of normally-sighted volunteers as well as from the QUT Optometry Clinic. The participants with glaucoma and the controls were recruited simultaneously and tested in order of recruitment.

Participants were excluded if they had any significant ocular or visual pathway disease leading to visual field loss, other than glaucoma; had Parkinson’s Disease; history of dizziness or vestibular disease; used a walking aid; or had signs of cognitive impairment (Mini-Mental State Examination score <24 of 30).[29] All participants were current drivers and licensed to drive in Australia, where the visual requirements are visual acuity better than 20/40 with one or both eyes and binocular visual fields with a horizontal extent of at least 110° within 10° above and below the horizontal midline.

The study adhered to the tenets of the Declaration of Helsinki and was approved by the Queensland University of Technology Human Research Ethics Committee. All participants were given a full explanation of the nature of the study and experimental procedures, and written informed consent was obtained. Participants attended two testing sessions including an assessment of visual function and self-reported driving, in addition to an assessment of on-road driving performance.

### Visual assessment and driving characteristics

All participants underwent an eye examination that included ophthalmoscopy, slit-lamp biomicroscopy, and fundus photography, to confirm eligibility for the study. Binocular visual acuity with the habitual driving correction was measured with the Early Treatment for Diabetic Retinopathy Study (ETDRS) chart at 5 m, at a luminance of 100 cd/m^2^, using the letter-by-letter scoring method.[30] Binocular contrast sensitivity was measured with the Pelli-Robson Contrast Sensitivity chart at 1 m at a luminance of 110 cd/m^2^, using the letter-by-letter scoring method.[31] A +1.00 DS lens was used to compensate for the working distance. Visual fields were assessed monocularly in each eye using the SITA-Standard 24–2 threshold strategy on a Humphrey Field Analyser (model 750, Carl Zeiss-Meditec, Dublin, CA). A binocular field was also constructed by combining the monocular visual fields based on the more sensitive of the two eyes at each visual field location.[32]

Participants also completed the Driving Habits Questionnaire (DHQ), an instrument used to characterize driving habits, exposure, frequency, driving difficulties and provide a self-rating of driving quality.[33] The degree of visual driving difficulty experienced in nine specific driving situations (when raining, driving alone, parallel parking, turning across on-coming traffic, highways, busy traffic, rush hour, night-time and unfamiliar areas), and a composite difficulty score scaled on a 100 point scale was generated,[33] where a higher score reflected less overall difficulty.

A brief general health questionnaire was administered to record the presence of 15 chronic medical conditions.[34] Hand grip strength was used as an index of physical frailty and assessed for both hands using a hydraulic hand dynamometer (North Coast Inc). The mean of the highest two measures of three trials (in kg-force) for the strongest hand was used in the analysis.

### Driving Performance

Driving performance was assessed under in-traffic conditions in an automatic, dual-brake vehicle using a previously validated technique.[35–37] An accredited professional driving instructor was responsible for directing the driver along the route, monitoring safety, and sat in the front passenger seat. Driving was scored by an occupational therapist seated in the back seat of the vehicle who is highly experienced in driving assessment and rehabilitation, having been involved in vision and driving studies for over 20 years; the agreement in ratings between the driving instructor and occupational therapist were high (intraclass correlation coefficient = 0.88, 95% CI 0.84–0.91, p<0.001), similar to previous studies.[36] The driving instructor and occupational therapist were both masked regarding whether the participants had glaucoma or not, and were also masked to participants’ functional performance in the laboratory testing. All assessments were conducted outside of rush hour traffic times.

Participants drove along a 19.4 km route on the open road, which started with a short familiarization period and then progressed to driving along city and suburban streets, and involved simple and complex intersections for a range of traffic densities. The driving assessment was around 50 minutes in duration, except when the drive was terminated early if the driver was considered too unsafe to proceed. A large proportion of the drive (~75%) was conducted under directed instruction, where the driving instructor provided detailed instructions of the route, while the remaining drive (~25%) was conducted under self-directed navigation, where the participant had to find their own way to a given destination. The occupational therapist scored driving performance at a series of locations along the driving route in terms of driving behaviors (at an average of 149 locations) and scored overall driving safety on a 10 point scale based on driving standards criteria described elsewhere.[37] The proportion of locations at which at least one error was made was also calculated.

#### Driving behaviors

At each of the locations, several aspects of driving behavior were scored: general observation (scanning and attention), braking/acceleration (appropriate speed and braking), lane positioning, gap selection (gap selected when entering traffic or the gap between the driver and other vehicles) and approach to hazards (appropriate planning and preparation).[38] Observation of blind-spots (correct checking of blindspot and shoulder checks) and indication/signalling (appropriate use of directional indicator) were also assessed where appropriate (average of 15 and 56 locations respectively). For each behavior type, the total number of errors as a proportion of the total number of times the behavior was assessed was calculated for each participant.

#### Driving situations

Each of the locations was further allocated into one of six situation categories: traffic light controlled intersections, one-way traffic (straight and curved driving), two-way traffic (straight and curved driving), give-way (stop/give-way intersections, non-traffic light controlled intersections, pedestrian crossings and roundabouts), manoeuvring (reversing, parking, turnaround manoeuvre and negotiation through traffic slowing devices), and merging (lane changing, merging and entering/exiting traffic flow). For each situation, the total number of errors as a proportion of the total number of times that performance was assessed at that location was calculated for each participant.

#### Critical Errors

Driving errors that were considered by the occupational therapist to pose a significant risk to driving safety and required an instructor intervention to avoid an imminent safety issue (either through applying the brakes or taking control/correction of the steering wheel) were classified as critical errors (CE). Each CE was coded post-testing into one of four broad themes which described the predominant factor associated with the CE: Observation (failed to observe key information in the driving environment); Speed control (excessive or very slow speeds); Lane control (drifting over lanes or failing to keep in lane), and Vehicle control (steering unsteadiness, problems with use of correct pedals or handbrake).

Where drives were terminated early because of unsafe performance, driving behaviors and situation errors were scored as a proportion of number of locations assessed, and overall driving performance was scored accordingly.

### Statistical Analysis

Group differences for the vision and self-reported driving characteristics were examined using independent t-tests and χ^2^ tests, where appropriate. Linear regression models were used to compare group differences in overall driving performance, driving behaviors and driving situations. For count outcome variables (CE and predominant CE factors), negative binomial regressions were used. All models were further adjusted for cognitive (MMSE score), motor (grip strength) and health status (number of co-morbidities) given that these factors have been shown to be associated with driving ability in previous research;[37] the inclusion of these potential confounding variables improved the statistical associations. Data were analysed with SPSS (ver. 22; www.ibm.com). Analyses were two-tailed, and p<0.05 was considered statistically significant.

## Results

Participants included 75 older drivers aged 65 years and above with a diagnosis of glaucoma and 70 visually normal controls; their demographic and visual characteristics are presented in Table 1. There were no significant between-group differences according to age, gender, number of co-morbidities, grip strength or cognitive function as measured by the MMSE. Visual function as measured by standard vision tests including visual acuity, contrast sensitivity and automated visual fields was significantly worse for the glaucoma patients than the controls, however, the between group differences for visual acuity and contrast sensitivity were small.

**Table 1:** Demographic and visual characteristics of the participants by group. Continuous variables presented in mean ± SD, with minimum to maximum values given for the vision variables.

	Glaucoma (n = 75)	Controls (n = 70)	p value
**Demographics**			
Age (years)	73.19 (5.95)	72.59 (4.99)	0.51
Gender (Female, n %)	23 (31%)	23 (33%)	0.78†
**Health**			
Co-morbidities (n)	2.65 (1.63)	2.69 (1.85)	0.91
Grip Strength (strongest hand: kg-force)	30.18 (9.60)	28.74 (9.82)	0.37
MMSE (score out of 30)	28.47 (1.48)	28.63 (1.19)	0.47
**Vision**			
Visual acuity (logMAR)	0.01 (0.11) (0.24 to -0.22)	-0.05 (0.09) (0.26 to -0.22)	<0.001
Contrast sensitivity (log units)	1.87 (0.14) (1.30 to 1.95)	1.95 (0.03) (1.85 to 2.05)	<0.001
Visual field MD, better eye (dB)	-1.21 (4.90) (-23.24 to 3.99)	1.86(1.15) (-1.89 to 4.25)	<0.001
Visual field MD, worse eye (dB)	-7.75 (8.47) (-31.00 to 2.10)	1.01 (1.38) (-2.98 to 3.54)	<0.001
Integrated visual field sensitivity (dB)	28. 86 (4.34) (8.67 to 34.04)	31.80 (1.30) (26.62 to 34.29)	<0.001

^**†**^ χ ^***2***^ ***test*,** *Independent Samples t-test used for continuous variables*, *and chi-square test for categorical variables*

Participants’ self-reported driving characteristics as determined with the DHQ are shown in Table 2. There were no between-group differences in terms of the number of days of driving in a typical week, estimated distance travelled per year, the spatial extent that a person drives in their own environment (Driving Space Score),[33] or the number of self-reported crashes. Very few drivers reported that someone had suggested they stop driving (1 glaucoma and 2 control drivers). When asked to rate the quality of their driving, the result was similar among the drivers with glaucoma compared to the drivers without glaucoma, with no drivers in either group reporting their driving as poor. Self-reported driving difficulties were, however, different between the groups, where the composite driving difficulty score for the glaucoma group was significantly worse than the controls (p = 0.005). However, further exploration of the specific driving situations where difficulties were reported indicated that the drivers with glaucoma reported significantly more driving difficulty for only two driving situations: driving when raining (p = 0.027) and at night (p<0.001). The remaining driving situations showed no differences between the glaucoma and control drivers (driving alone, p = 0.98; parallel parking, p = 0.96; turns across oncoming traffic, p = 0.21; driving on highways/motorways, p = 0.81; high-traffic roads, p = 0.14; rush hour, p = 0.31; and unfamiliar areas p = 0.18).

**Table 2:** Self-reported driving characteristics of the participants as determined from DHQ responses.

Characteristic		Glaucoma (n = 75)	Controls (n = 70)	P value^*†*^
Years of driving experience (mean (SD))		54.6 (6.3)	53.4 (7.1)	0.29
Distance driven in the past year (n, %)	< 5000 km	14 (19)	20 (28)	0.28
	5001–10,000 km	39 (52)	26 (37)	
	10,001–15,000 km	9 (12)	14 (20)	
	15,001–20,000 km	7 (9)	6 (9)	
	>20,000 km	6 (8)	4 (6)	
Number of days driven in a typical week (mean (SD))		4.9 (1.9)	5.3 (1.7)	0.16
Composite Driving Difficulty Score (mean (SD))**		92 (10)	96 (6)	0.005
Drive Space Score, (mean (SD))***		18.2 (8.5)	17.7 (7.2)	0.72
Someone has suggested stopping driving (n, %)		1 (1.3)	2 (2.9)	0.52
Driver Dependency score (mean (SD))^		1.61 (0.70)	1.54 (0.72)	0.63
Quality of Driving (n, %)	Excellent	8 (11)	5 (7)	0.42
	Good	46 (62)	49 (70)	
	Average	18 (24)	16 (23)	
	Fair	2 (3)	0 (0)	
No. of drivers with a history of crashes (n, %)	In previous 12 months	3 (4)	4 (6)	0.64
	In previous 5 years	14 (19)	16 (23)	0.56

^†^ Independent Samples t-test used for continuous variables, and chi-square test for categorical variables.

** Out of 100; lower scores indicate greater difficulty

*** Summative score of driving patterns away from their home base over the past 6 months (e.g., within the neighborhood, outside the state). Lower scores indicate less frequent and smaller driving space.

^ Ranges from 1–3 with higher scores reflecting greater levels of dependency on others to drive.

The on-road driving characteristics of the drivers with glaucoma and controls are presented in Table 3. The glaucoma drivers were rated as less safe overall than the controls, with a 0.62 point difference (5.17 vs. 5.79, respectively), and made more errors overall relative to opportunity than did the control drivers (16% vs 14%, respectively); these differences reached significance after adjustment for cognitive status, strength and health status. Adjusting the model for years of driving experience did not affect the outcome. Negative binomial regression with adjustment indicated that the average number of CE errors per drive was significantly higher for the glaucoma than the control drivers (0.83 vs 0.43, respectively; rate ratio (RR) 2.06, 95% CI 1.17–3.62). When the predominant CE factors were examined, the rate of CE relating to observation in glaucoma drivers was significantly higher than in controls (RR 2.06, 95% CI 1.04–4.08), while no differences were found for CEs relating to vehicle control, lane discipline or speed. The driving assessment was terminated early due to safety concerns in 12 drivers; the proportion did not differ between the groups (glaucoma: n = 6 (8.0%) vs controls: n = 6 (8.6%), χ_*1*_^***2***^ = 0.016; p = 0.90).

**Table 3:** Summary scores for on-road driving assessment as a function of group status (mean ± SD) with unadjusted and adjusted p values.

Driving Outcome	Glaucoma (n = 75)	Controls (n = 70)	Unadjusted p value	Adjusted p value†
Driver safety rating (1–10)^a^	5.17 (1.81)	5.79 (1.96)	0.05	0.028*
Proportion of locations with an error (%)^a^	16 (6)	14 (5)	0.057	0.041*
Critical errors (CE) (#)^b^	0.83 (1.16)	0.43 (0.73)	0.02*	0.012*
CE relating to Observation (#)^b^	0.48 (0.76)	0.24 (0.52)	0.044*	0.037*
CE relating to Vehicle Control (#)^b^	0.11 (0.35)	0.14 (0.39)	0.561	0.597
CE relating to Lane Discipline (#)^b^	0.19 (0.39)	0.16 (0.40)	0.693	0.554
CE relating to Speed (#)^b^	0.15 (0.56)	0.06 (0.29)	0.120	0.079

† Adjusted for MMSE score, grip strength and number of co-morbidities

* p<0.05

^a^ Linear regression

^b^ Negative binomial

Blind-spot errors were the most common form of driving behaviour errors in both groups, however, there were no significant between group differences (Table 4). The drivers with glaucoma made significantly more errors associated with approach (p = 0.016), observation (p = 0.013) and appropriate lane position than the controls (p = 0.011). Of the driving situations, most errors were made in merging situations, but there were no significant between group differences. However, drivers with glaucoma made significantly more errors at traffic light controlled intersections (p = 0.005) and give way situations (p = 0.039) compared to controls.

**Table 4:** Specific error types and locations of errors made as a function of group.

	Glaucoma (n = 75)	Controls (n = 70)	Unadjusted p value	Adjusted p value†
Driving behaviours (% errors to locations assessed)				
Observation errors	3.79 (3.41)	2.94 (2.56)	0.091	0.013*
Brake/accelerator	5.10 (5.07)	4.26 (4.08)	0.278	0.228
Indicator	11.40 (5.44)	10.69 (4.81)	0.407	0.390
Lane position	6.73 (4.97)	4.98 (3.92)	0.020*	0.011*
Gap selection	2.04 (1.59)	1.76 (1.77)	0.314	0.261
Approach	6.27 (5.27)	4.46 (4.19)	0.024*	0.016*
Blindspot	74.23 (19.78)	69.29 (19.59)	0.133	0.104
Driving situations (% errors to locations assessed)				
Traffic Light Situations	13.53 (9.72)	9.41 (8.18)	0.007**	0.005**
One-way driving	17.94 (17.33)	16.90 (18.20)	0.725	0.672
Two-way driving	13.76 (9.98)	11.06 (8.98)	0.090	0.077
Give-way	24.12 (11.03)	20.97 (8.90)	0.061	0.039*
Manoeuvring	35.58 (21.79)	41.58 (21.26)	0.096	0.118
Merging	66.33 (17.72)	64.67 (16.59)	0.560	0.469

† Adjusted for MMSE score, grip strength and number of co-morbidities

* p<0.05

** p<0.01

## Discussion

This study investigated which types of driving errors and driving situations are more problematic in a large sample of drivers with mild to moderate glaucomatous field loss compared to control drivers without glaucoma. Our data reveal that even drivers with mild to moderate field loss exhibit impaired driving performance compared to age-matched controls, being rated as less safe than the controls, having significantly more problems with observation, maintaining lane position and approach and planning than the controls, with driving errors being more common at traffic-light controlled or give-way intersections. The drivers with glaucoma were also significantly more likely to make one or more critical errors that required the driving instructor to intervene and these interventions were particularly associated with errors in observation. This finding that even mild to moderate glaucomatous field loss has important functional consequences supports other research that has shown impairments in activities of daily living such as eye-hand coordination [39], balance and falls [40, 41] even in those with modest glaucomatous field loss.

The types of driving errors made more often by the glaucomatous drivers included maintaining lane position, approach (including changing lanes and planning ahead) and observation, which suggest problems in appropriately observing the road environment and planning ahead. The finding of increased lane position errors supports the findings of the two small sample studies of drivers with binocular glaucomatous field loss,[26, 42] which reported lane keeping as one of the reasons that these drivers failed an on-road assessment, while the finding of problems with accurate observation of the road ahead in complex road situations has not been previously investigated. Maintaining appropriate lane position and changing lanes requires awareness of peripheral hazards in the driving environment, as well as those directly in front of the driver and may reflect reductions in peripheral awareness. However, not all of the drivers with glaucomatous field loss exhibited these driving errors suggesting that some drivers are able to compensate for their field loss. This is supported by previous studies of drivers with binocular hemianopic [42, 43] and glaucomatous field loss, [25, 42, 44] where some drivers exhibited differences in visual scanning and eye movement patterns compared to controls, particularly towards the area of their visual field defect; alternatively, some drivers may slow down and adopt a more cautious driving style to compensate for their impairment. While we did not assess eye movements or scanning patterns in this study, it is an important area for future research in order to enable better understanding of whether certain patterns of eye movements can compensate for visual impairment; these strategies could be incorporated into training scanning techniques for those with visual impairment with potential improvements in driving safety.

The drivers with glaucoma made significantly more errors than controls at traffic light-controlled and give-way (yield) intersections. Importantly, the consequences of errors in these traffic situations are likely to involve multiple road users (vehicles and pedestrians) and have significant safety consequences. While crash data suggests that increasing age is a significant risk factor for the prevalence and severity of intersection crashes,[45] this is the first study to identify intersections as a particular performance problem area for drivers with glaucoma.

The finding of self-reported problems with night-time driving concur with those of a large study by Janz et al[6] and other studies that indicate that problems with driving at night are a common complaint in patients with various forms of visual impairments, including cataracts, glaucoma and age-related macular degeneration,[6, 7, 33, 46] as well as in patients following refractive surgery and those wearing presbyopic corrections.[47, 48] However, there were few self-reported differences in driving habits or self-rating of driving quality between the drivers with and without glaucoma. This finding is in contrast with the significant differences evident in the assessment of on-road driving performance which was undertaken in relatively good driving conditions, being in daytime hours and when the weather was dry. Many of the drivers in both groups, but particularly the drivers with glaucoma, had critical errors that required an instructor intervention and had correspondingly poor ratings of overall driving safety, yet no participants rated their driving as poor and very few drivers had been advised to stop driving. Interestingly, other studies of older drivers in general,[49] and specifically those with hemianopic field defects[50] suggest that lack of insight is a problem in some drivers. Therefore it is imperative that objective predictor tests are identified that can predict those drivers who are unsafe to drive, as it is clear that some drivers are less insightful regarding their driving capacity.

The results of this study should be considered in terms of both its strengths and limitations. Strengths include a driving assessment under real traffic conditions in a wide variety of on-road environments using a standardized route that was both extensive in duration and length and included a variety of different driving challenges which allowed determination of both the type of errors and also the type of traffic locations that were more problematic. Furthermore, both the occupational therapist and driving instructor were masked to participants’ visual characteristics and whether they had glaucoma or not. Finally, we assessed drivers with a range of glaucomatous loss, from early to moderate loss, as these older adults are most likely to be driving on normal road systems, in addition to a representative sample of control drivers without glaucoma. A limitation, as in any study of this nature, is that the drivers were aware that they were being assessed and may have adjusted their driving performance accordingly. In addition, drivers were also assessed in an unfamiliar vehicle and in an unfamiliar driving environment that may have impacted on compensatory strategies; naturalistic studies of drivers with glaucoma in their own vehicles and in their own environments are therefore important for future research, given that many drivers with visual impairment are given *conditional* licences that permit them to drive only within a limited radius of their home and/or during daylight hours. As for any study conducted under in-traffic conditions, there may be minor variations in weather and traffic, although assessments were only conducted in dry conditions and outside of peak hour traffic, and the occupational therapist took these variations into consideration when rating participants’ driving performance. Indeed, the fact that the study was also conducted outside of peak hour traffic may underestimate the potential driving problems of some of the participants when driving in busy traffic situations.

In summary, this is the first on-road study to include a large sample of drivers with glaucoma enabling identification of the specific driving error types and driving locations that are most problematic for drivers with glaucoma compared to control drivers without glaucoma. We demonstrated that older drivers with mild to moderate glaucomatous field loss exhibited less safe overall driving performance, and made more errors involving specific driving behaviours and situations. Importantly, in a growing population of drivers with mild to moderate glaucoma, the impact of these differences on road safety will be critical, particularly as problem areas include situations such as traffic-light controlled intersections, where the consequences of errors can be fatal. Thus for older drivers with glaucoma early detection is important, not just to minimise or prevent progression of disease and its associated visual impairment, but also to ensure safe driving performance. We will explore the relationship between a range of standard and novel visual function tests and the driving outcome measures and safety in future papers, which will assist in the design of test batteries to better identify unsafe drivers. Future research will also use these results as a basis to explore potential interventions that can improve the driving ability and safety of this growing population.
